# A Real-Time Epilepsy Detection Method Using Embedded Zero Tree Wavelet Approach and Support Vector Machine

**DOI:** 10.1155/bn/5916201

**Published:** 2025-08-26

**Authors:** P. Padmapriya, V. Rajamani

**Affiliations:** ^1^Department of Biomedical Engineering, SRM Institute of Science and Technology (Deemed to Be University), Ramapuram Campus, Chennai, Tamil Nadu, India; ^2^Department of Electronics and Communication Engineering, Chettinad Academy of Research and Education, Manamai Campus, Chennai, Tamil Nadu, India

**Keywords:** electroencephalogram (EEG), embedded zero tree wavelet (EZW), epilepsy, support vector machine (SVM)

## Abstract

Temporary disturbances in brain function are caused by epilepsy, a chronic disorder resulting from sudden abnormal firing of brain neurons. This research introduces an innovative real-time methodology representing detecting epileptic spasms from electroencephalogram (EEG) data. It employs a support vector machine (SVM) alongside embedded zero tree wavelet (EZW) transform. To facilitate precise multiresolution analysis of epileptic convulsions, the EZW method is selected for its capacity to efficiently compress multichannel EEG data while preserving crucial diagnostic features. EZW effectively captures and encodes key patterns in EEG signals, enabling detailed analysis of the subtle variations associated with seizures. This study extracts statistical features such as entropy, kurtosis, skewness, and mean from the compressed EEG segments. These features are then classified using the SVM to distinguish between normal and epileptic states. With a remarkable 99.02% classification accuracy and a false positive rate of only 1.1%, the proposed algorithm demonstrates excellent performance. The novelty lies in integrating SVM with EZW-based feature extraction and advanced preprocessing, enabling efficient real-time EEG analysis. Unlike previous works, this approach preserves critical information, enhances classification accuracy, and supports multichannel signals, offering a robust and practical solution for real-time epilepsy detection. Based on these findings, the method is considered highly suitable for real-time implementation in clinical environments.

## 1. Introduction

The brain controls emotional and neurological processes; it is the body's most active organ. The brain's electrical impulses are monitored and shown graphically utilizing EEG, a commonly used electronic device in engineering, neurological disorders, microelectronics, bioengineering, and neurophysiology [1]. Two types of EEG exist: intracranial, in which electrodes are surgically implanted into the brain, and scalp, in which tiny electrodes are mechanically and electrically attached to the scalp. A variety of brain illnesses, including autism and epilepsy, can be brought on by variations in electrical activity. EEG is used to identify other aberrant brain states, monitor cognitive tasks, analyze sleep patterns, and diagnose illnesses, including epilepsy and seizures [2]. Biomedical signal compression is required because the EEG signal requires a large amount of storage and transmission capacity due to its multichannel nature. For low compression ratios, lossless compression is the way to go. On the other hand, lossy compression is more often used since it works and relies on the signal's distortion at a certain compression ratio [3, 4]. ECG signals were processed using embedded zero tree wavelet (EWZ) data compression in the work of Shapiro. This method successfully encodes wavelet packet coefficients from various subbands within an audio stream. In a later publication, the authors utilized wavelet transforms to create the zero tree wavelet method [5, 6]. In this case, the signal is broken down into several bands, with the low-frequency bands being crucial to the reconstruction process. The rate of compression and PSNR (peak signal-to-noise ratio) have been increased substantially as part of the H.H. band coefficients are deemed irrelevant.

Disruptions in the normal electrical activity of the brain are the root cause of seizures, impacting around 50 million individuals globally. The repeated and unpredicted seizures that characterize epilepsy are classified as a chronic condition. Under some circumstances, it may cause unconsciousness, and the person may not remember the incident [7–9]. Interictal activity, or the interval between episodes, and ictal activity, or the interval between seizures, are the two separate groups that epilepsy shows in EEG signal recordings [10]. Initiating antiepileptic drug therapy is contingent upon receiving a prompt and precise diagnosis of epilepsy since this mitigates the likelihood of further seizures and related problems [11]. Usually, manual methods are used by neurologists and other medical experts to identify these illnesses, mostly through visual EEG analysis, which takes a lot of time and is less efficient. Various useful diagnostic techniques have been developed to aid in diagnosing and treating abnormalities and behavioral issues, attention deficits, learning difficulties, and language delays [12]. Selvam et al. developed an automated EEG monitoring system to identify epileptic seizures that used the spectrum and frequency domain features to identify brain cancer [13]. FIR and IIR+ FNN-GRU are two filtering techniques that Bouallegue et al. suggested to extract various characteristics from intricate EEG data recordings [14].

Through the selection of pertinent subbands, this technique increases diagnostic efficiency. CNNs used ICA to achieve 99% accuracy for seizures in the Bonn and MIT datasets and 99.5% accuracy on the KAU dataset. Multiwavelet transformation is used to break down EEG signals in order to extract features, and approximation entropy is used to identify anomalies. A feedforward neural network has an 87% classification accuracy for brain tumors and a 93% classification accuracy for first-level epileptic episodes [15, 16]. After preprocessing the EEG data to remove artefacts, Ibrahim et al. utilized DWT to divide the data into subbands. Nonlinear techniques evaluated EEG complexity in addition to Lyapunov exponent and Shannon entropy. 94.6% accuracy was attained in a three-class classification using KNN with DWT and Shannon entropy [17]. Bhattacharyya et al. provided a real-time seizure identification technique based on an empirical wavelet transform approach [18]. In an alternative study, Bomela et al. employed the Fourier approach to identify the network link for seizure start detection. They achieved a 0.16 rate of false positive over an hour (FP/h) and a 93.6% sensitivity [19]. A technique that combines the relevance vector machine and harmonic wavelet packet transform was presented by Vidyaratne et al. [20].

Recent studies have increasingly adopted hybrid and adaptive learning methods to enhance EEG-based seizure detection. A notable example is the use of a genetic algorithm–assisted fuzzy Q-learning classifier that adaptively learns seizure patterns, enhancing model accuracy and resilience [21]. Another prominent work uses fuzzy lattices with neural reinforcement learning to effectively capture uncertainty and nonlinear behavior in EEG data, achieving robust classification results [22]. Wavelet transform remains integral in EEG feature extraction. Albaqami et al. [23] proposed a dual-tree complex wavelet model for multiclass seizure detection and reported accuracies above 99% across benchmark datasets. Shen et al. [24] introduced a real-time seizure detection method combining tunable-Q wavelet transforms with CNNs, demonstrating over 96% accuracy and high sensitivity. Moreover, a novel method combining wavelet-based feature fusion with deep learning classifiers was reported to outperform traditional models, achieving state-of-the-art detection metrics on large-scale datasets [25]. These approaches reflect a shift toward adaptive and high-precision models, reinforcing the relevance of wavelet-based frameworks such as EZW used in the present study.

Spike and sharp movement are the key EEG abnormalities detected during epileptic episodes [26]. Examining feature extraction techniques aids in determining the eigenvalues that distinguish regular waves from spike or sharp waves during seizure phases. TQWT, MAS, EMD, DWT, Q-wavelet transformation, and HHT are among the methods used in the frequency, time, and time–frequency domains [18, 27–31]. Machine learning has greatly improved epileptic seizure identification. Studies have classified seizure states using SVM, random forests, logistic regression, naive Bayes, and LDA [27, 31–35]. Automated epilepsy detection includes offline and real-time methods. Offline detection distinguishes seizures from EEG data, while real-time detection minimizes delays during continuous monitoring. Using DWT with RUS boosted tree ensemble, Shen et al. attained sensitivity of 96.15%, accuracy of 96.38%, and a false positive (FP) rate of 3.24% [11, 36, 37]. For real-time seizure detection, Boston Children's Hospital CHB-MIT dataset was utilized by the researchers. Samiee et al. achieved 70.19% sensitivity using multivariate textural features and GLCM in SVM [38]. Zarei and Asl used nonlinear features with SVM and orthogonal matching pursuit with DWT for seizure detection. Other studies have applied graph theory, functional connectivity, and effective connectivity analysis [27, 39–41]. Zarei and Asl used orthogonal matching pursuit with DWT for seizure detection, integrating nonlinear features with an SVM classifier. Their FP rate was 2.74%, while their sensitivity was 96.81% [42]. Li et al. developed a model combining SVM, EMD, and similar spatiotemporal patterns, achieving sensitivity of 97.34% besides a FP rate of 2.5% [43]. This investigation evaluates the effectiveness of several methods for improving MRI image estimation and presents a novel methodology called the no-reference image quality index. A novel approach called optimized deep knowledge–based NIQI (ODK-NIQI) has also been created and tested [44]. The proposed strategy demonstrates the highest consistency and performance in noisy and denoised MRI brain images. To improve seizure detection, this paper introduces a novel framework, addressing the time-sensitive and uncertain outcomes of traditional manual EEG signal assessment by medical practitioners [45, 46]. Using TF domain machine learning, Dasha et al. presented a method for epilepsy identification based on EEG signals captured by wearable sensors during various forms of physical exercise [47]. In order to train ML and DL models, we first use GWST to get the TF domain representation of the EEG data, and then, we extract entropy and L1-norm features. With hold-out validation, the random forest model can diagnose epilepsy with an accuracy of 90.74%, while the multilayer LSTM model achieves an accuracy of 87.04%. For activity-specific leave-one-out cross-validation, multilayer LSTM achieves accuracies of 88.89% and 70.83% for walking and running [47]. Liu et al. introduced a weighted normalized entropy–based criterion for selecting TQWT parameters to better capture EEG oscillations. They used refined TQWT parameters to decompose EEG signals, removing redundant information and highlighting epileptic features. The RTQWT and decision tree combination achieved the best accuracy, improving by 4.8% to 18.6% over other methods, with an accuracy of over 95% on four datasets, and XGBoost performed best with 89% accuracy on the AB-CD-E dataset [48]. The study contains seven separate classification tasks for accurately identifying epileptic episodes. Wireless telemonitoring from the receiver side may benefit from the proposed method, which has been tested with AWGN at various signal-to-noise ratio (SNR) levels [49]. The FBSE-EWT decomposes EEG signals into subbands, followed by a 3D phase space reconstruction (PSR) to calculate entropy-based features like LL, LEEnt, and NEnt. To categorize EEG signals from epileptic seizures, the Kruskal–Wallis test scores these characteristics, and top classifiers including RF, ET, xgBT, B-SVM, and B-k-NN are employed [50]. Sharma et al. used the IEVDHM method to decompose nonstationary signals and applied Hilbert transform to derive the time–frequency representation (TFR). The method, evaluated on synthetic signals, showed superior performance in classifying epileptic seizures using LS-SVM with 100% accuracy [51]. Additionally, the FLHF method and FD analysis achieved 99% sensitivity, 99.5% specificity, and 99% accuracy using GBSO-TAENN classifier, outperforming other approaches [52]. Sharma et al. demonstrated the benefits of iterative filtering (IF) over empirical mode decomposition in classifying seizure-related EEG signals. Using features from amplitude envelope functions and intrinsic mode functions, a random forest classifier was trained to achieve excellent classification accuracy while drastically lowering the processing duration for real-time applications [53]. EMD models AM-FM signals, which are used to divide EEG signals into IMFs. To improve the accuracy of seizure event categorization, the Hilbert transform is employed to create bandwidth characteristics (BAM and BFM), which are then used in an LS-SVM [54].

Numerous research investigations have explored various techniques for EEG-based epilepsy detection, but the effectiveness of such systems is often determined by their accuracy and resilience. Many existing studies have produced results that fall short of expectations due to the inherent challenges in handling EEG signals. EEG signals are continuously captured over long periods, with each sample containing critical subject-specific information. However, many systems fail to preserve this essential information during compression, particularly when dealing with multichannel EEG signals. This loss of key data undermines the utility of the system for specialists who rely on accurate and complete information to make diagnoses. To address these challenges, the present research focuses on designing a system that can efficiently diagnose epileptic diseases using real-time EEG data while ensuring the preservation of critical information during compression. This approach emphasizes the development of a superior feature extraction method that allows for efficient compression of large quantities of multichannel EEG signals without losing important details. By doing so, the system is aimed at assisting neurologists in diagnosing neurological brain problems more effectively. Examining multiple feature extraction as well as classification methods, this study seeks to identify the optimal strategy to analyze EEG data for the purpose of diagnosing epilepsy. In preprocessing, histogram equalization is applied to the EEG data to enhance its quality by adjusting the contrast and highlighting the relevant elements. Important feature extraction follows this preliminary preprocessing using the EZW method. This method efficiently compresses multichannel EEG data by combining various statistical methodologies, including variance, kurtosis, entropy, mean, and standard deviation (SD). These features help preserve essential information while reducing the overall data size. To classify the extracted features and perform in-depth analysis, SVM classifier stands employed. Through learning from the features that have been extracted and using the data to make accurate predictions, this classifier helps to differentiate between events that are associated with epilepsy and those that are not. The important contributions of this paper are as follows:

• Development of an advanced system that uses real-time EEG data for quick and accurate diagnosis of epilepsy.
• Introduction of improved feature extraction method using the EZW for compressing multichannel EEG data without losing crucial information.
• Use of histogram equalization during preprocessing to enhance the quality of EEG signals.
• Application of SVM classification for in-depth analysis and accurate categorization of EEG signals.
• The overall aim is to help neurologists diagnose neurological disorders more effectively by providing a robust and efficient system for analyzing EEG data.

A transparent and rational development of the study is provided by the document's organization, beginning with the gathering of data and ending with the results. The following is the organization.

Section 2: The EEG data used in the study is thoroughly explained in this section, along with the methods used for data processing and analysis. It covers crucial procedures like preprocessing, which prepares raw data for analysis, signal processing, and filtering methods to improve signal quality. The section ends with a summary of the classification strategies used to successfully classify the signals. It also covers feature extraction techniques, including how important information is extracted from the EEG data.

Section 3: It is in this part that the analysis presented in Section 2 is displayed. The thorough analysis of the outcomes emphasizes the efficiency and efficacy of the proposed approaches. This section analyzes how well the feature extraction and classification processes worked under different circumstances and assesses how well the techniques detected seizures.

Section 4: Based on the findings covered in the preceding section, the final section requires a succinct outline of the study's contributions. Additionally, it highlights areas that could be further investigated or enhanced to improve the system's overall performance and applicability in real-world scenarios, suggesting possible directions for future research.

## 2. Methods

**Figure 1:**
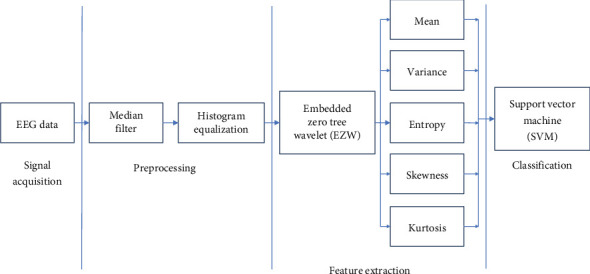
Schematic representation of the proposed system.

This section offers a thorough explanation of the proposed feature extraction, classification, and validation processes, implemented using MATLAB software. The method and the EZW-based techniques are visually represented concerning Figure 1. As seen in Figure 1, the first step of the process is to gather EEG data. Then, to improve the signal quality, artefacts are removed using a histogram equalization approach. Upon successful removal of the artefacts, the EEG data is segmented into smaller, manageable parts. The segments are subsequently subjected to the EZW technique for feature extraction. We can derive important statistical properties from EEG signals, including entropy, variance, kurtosis, SD, and mean. Feature vectors, which are organized sets of extracted features, are essential for improving the classification system's performance. The application of EZW ensures the effective handling of multichannel EEG data while preserving important signal characteristics that are essential for accurate classification.

### 2.1. Experimental Procedure and Data Acquisition System or EEG Data Collection

**Figure 2:**
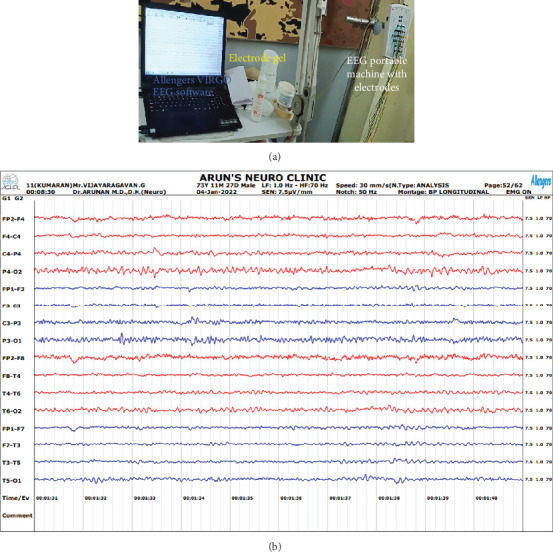
(a, b) Real-time EEG recording (normal subject) with Allengers EEG machine.

**Figure 3:**
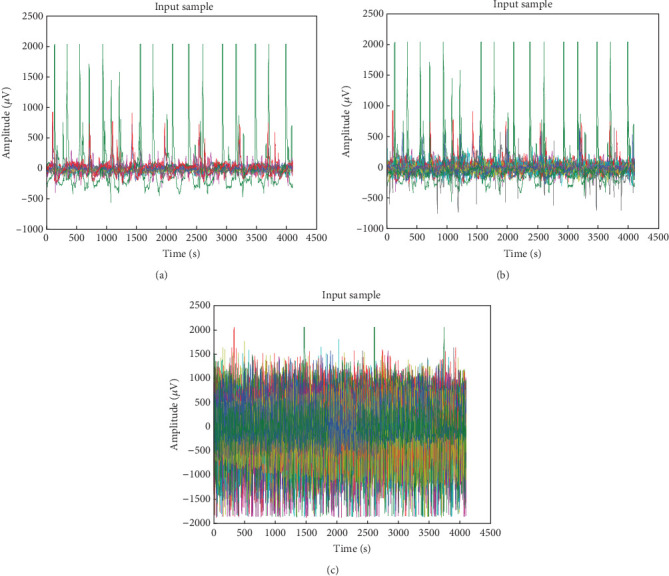
(a–c) Input EEG signal obtained through various channels from the human brain's scalp, 1 channel, 10–25 channels, or 50 channels.

The EEG data from numerous participants of normal and epilepsy subjects was obtained using the EEG VIRGO 24, VIR211160406 model, which was equipped with a preset amplitude-based DSA bar realigned configuration. The data collection was conducted in collaboration with Arun's Neuro Clinic, India, where normal and epilepsy subjects aged between 12 and 65 years were included. The institutional ethical committee (SRMIST-RMP/Ethical Committee/2023/023) reviewed the request and gave its stamp of approval. Prior to participating in this study, all individuals gave their written informed permission. During calibration, baseline wandering processing was performed using a BLC filter. When the system was first set up, the low-pass filter was set to 1.0 Hz, the high-pass was set to 70 Hz, the notch was set to 50 Hz, the input sensitivity was set to roughly 7.5 *μ*V/mm, and the recording speed was set to 30 mm/s. The study used EEG data from 500 individuals with epilepsy and 400 individuals with normal behavior. With or without their eyes open, the subjects' EEG data were recorded at a 173 Hz sampling rate and a 0.5–85 Hz spectral spread. After lying in a comfortable posture, the individual will be given gel to apply in several places where the electrodes will be placed. According to the doctor's advice, the electrodes are precisely placed to reduce interference from impedance matching, and signal recording is carried out for around an hour and 50 min. Data collection includes time-series measurements of segmented electrical brain waves from individuals with epilepsy.  Figure 2 illustrates the procedure used to get data from signals from an EEG under typical circumstances, where all channels exhibit regular brain waves. In an effort to improve acquisition efficiency, Figure 3 shows the input of EEG signals obtained for the same individual from various channels, including single-channel, 10–25 channels, and 50–100 channels. Each channel's EEG data is displayed using a distinct color scheme. Compared to data collected using many channels, EEG signals in a single channel are less noisy.  Table 1 compiles the performance of other peer works in seizure detection using various datasets. Unlike previous studies, this research utilized all 15 datasets.

### 2.2. Preprocessing of the Input EEG Signal

Three signal preprocessing processes comprised equalization, filtering, and artefact elimination. EEG signal artefacts may arise from various external sources, including respiration, limb movement, blood pressure, electricity lines, magnets, and mobile wave noise. A reference signal of four electrodes was put around the eyes to minimize interference from blinks while data was being collected. Following partitioning the EEG dataset into equal-sized parts, a 50-s sample was chosen to enhance preliminary processing, SNR, and accuracy [44]. The application of linear adaptive histogram equalization was used to improve contrast and distribute pixel values uniformly. A popular method for improving image contrast is HE; it modifies the image based on the sample distribution. This produces a smooth cumulative histogram and a uniform pixel value distribution.

### 2.3. EZW for EEG Feature Extraction

The EZW encoder processes both signals and images as two-dimensional data by applying wavelet transform. This method utilizes gradual or embedded encoding, which effectively reduces reconstruction errors while compressing the data into a bitstream. In addition, this approach ensures data integrity by preventing any potential loss during the encoding and compression processes. When WT is used on pictures, the subband energy decreases with increasing dimensional scale, meaning that increased resolution is associated with lower scale. This results in the wavelet coefficients in the higher bands being generally reduced compared to those in the lower subbands [52]. For EEG data, however, a 1-D WT approach is employed, where each subband consists of both high-pass and low-pass components, which grants more accurate interpretation of the signal's characteristics. The EZW algorithm performs a downsampling process that progressively reduces the length of the detailed substrips and core by half at each scale level, compared to the initial level. The reconstruction of the time–domain output properly requires a downsampling approach that maintains every segment of the original signal while keeping the total quantity of WT coefficients constant. This approach balances compression with the retention of key signal details, making it highly effective for efficient data representation and reconstruction.

#### 2.3.1. Zero Tree Wavelet Encoder Embedded

**Figure 4:**
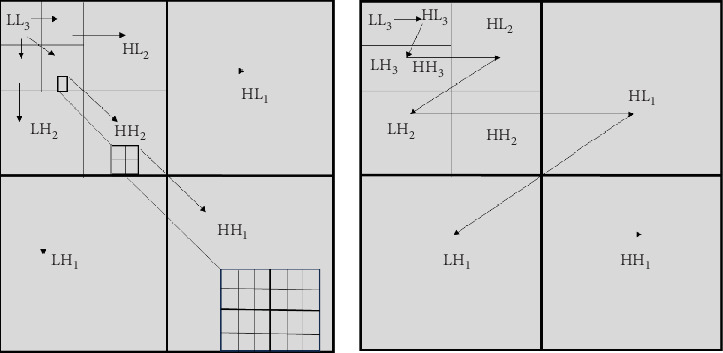
Subband order in a three-level wavelet decomposition using wavelet tree scanning.

The WT used to encode and compare coefficients using the *T*-threshold technique, which differs depending on the pass. Refineries and dominant were the two pass categories. If, after encoding and processing, the coefficient in the predominant case has a value larger than *T*, the subsequent step was changed. Otherwise, as a minimum signal in the process, it was skipped for encoding if it did not exceed *T*. Following completion of scanning, the *T*-threshold was divided into equal segments once more and subjected to data scanning in the subsequent run.  Figure 4 depicts a three-level decomposition of wavelet procedure using the wavelet tree and the subband scanning order. This process was repeated until larger values were efficiently encoded, depending on the null tree approach [53, 55].

#### 2.3.2. Zero Tree Wavelet Decoder Integrated

**Figure 5:**
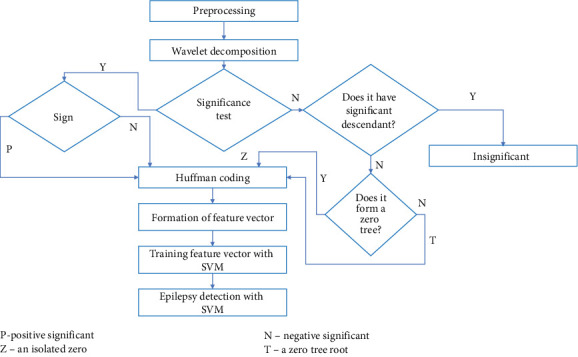
The process of the feature extraction by EZW.

As shown in Figure 5, the entire process of EZW feature extraction is illustrated. The threshold value, *A*_ezw_, is first retrieved by the EZW decoder according to Equation (1). This value is then used to fill the empty coefficient table with 0 and other values. Whether *T* was more significant, smaller, or zero bits in the entering bit stream of the pass determines the value in the table. The current value and its children's values are set to zero upon receiving a zero tree phrase. Each monitored coefficient's current value is assessed and modified correspondingly for nonzero values. *A*_ezw_ is cut in half and repeated as the next batch of dominant pass data is examined. Where there are a few null coefficients, the coefficients are changed to null, and the new *A*_ezw_ value may be added. Every coefficient is accurately encoded and then entirely rebuilt by the decoder if the encoder operation is not stopped. Channel differential coding has been used instead of separately coding each channel since EEG records have several timestamps. The graphic shows the EZW method for extracting signal characteristics. The signal is band-pass-filtered using the wavelet algorithm. Next, the reconstructed signal is assessed to determine relevant and irrelevant information. The feature vector is created correctly, and coding is applied if the signal shows significant components. Different characteristics are derived from these feature vectors and used in training and testing.

Assume the following discrete-time presentation of the EEG signal:

(1) $$\begin{array}{c} A_{\text{ezw}}\left(l,m\right)\text{: }l\in 1\cdots Z_{\text{eeg}},m\in 1\cdots p_{\text{eeg}}\times q_{\text{eeg}} \end{array}$$

where *q*_eeg_ is the EEG signal recording quantity, *p*_eeg_ speed (seconds) is the duration of the EEG signal capture, and *Z*_eeg_ (Hertz) is the frequency of signal sampling.

A set of *N* samples from the *l*-th channel on a temporal scale takes the form of a vector *V*(*x*), as shown in Equation (2). These vectors are treated as components of the *l*-th channel. Instead of using sl_x1_ and sl_x2_, two vectors collected at the same time and associated assume a_x1_ and a_x2_, for encoding. The correlation between these vectors is evaluated using the correlation coefficient, and similarity metrics between segments are calculated based on signal measures, such as the infinity error norm of the peak or the L2 error norm of the energy signals. More sophisticated correlation measures, like the angle between the sequence vectors of the time–domain data, are also investigated. When l_x1_ and l_x2_ represent the angles derived from al_x1_(*i*) and al_x2_(*i*), respectively, the angle between them, denoted as ∅_l_x1_ and l_x2__(*i*), is computed using the inner product operator. This method provides a refined way to assess the relationship between different signal components:

(2) $$\begin{array}{c} V\left(x\right)≔A_{\text{ezw}}\;\left(q_{\text{eeg}},\left(x-1\right)N+1\cdots ij\right),q_{\text{eeg}}\in 1\cdots q_{\text{eeg}},y\in 1\cdots \frac{p_{\text{eeg}}\cdot z_{\text{eeg}}}{N}, \end{array}$$

(3) $$\begin{array}{c} \emptyset_{{\text{l}}_{\text{x}1},{\text{l}}_{\text{x}2}}\left(i\right)=\frac{{\text{al}}_{\text{x}1}\;\left(i\right)\cdot {\text{al}}_{\text{x}2}\;\left(i\right)}{\left|{\text{al}}_{\text{x}1}\;\left(i\right)\right|\;\left|{\text{al}}_{\text{x}2}\;\left(i\right)\right|\;}. \end{array}$$

#### 2.3.3. Significance and Operation of EZW in EEG Signal Analysis

The EZW is a highly efficient wavelet-based compression and feature extraction method that enables both dimensionality reduction and retention of key signal characteristics. Originally developed for image compression, EZW operates on the principle of progressive encoding, where coefficients are encoded in a hierarchical, tree-like structure known as zero trees. These zero trees capture the spatial and frequency correlations across wavelet subbands, allowing the algorithm to efficiently discard insignificant coefficients (noise or noninformative parts) and focus on encoding only the dominant features. In the context of EEG signal processing, the EZW algorithm decomposes time-series signals using 1D wavelet transforms into subbands that represent various frequency components. Each subband includes high-pass and low-pass filtered versions of the signal, effectively localizing both time and frequency information. The coefficients obtained from this decomposition are then organized into zero tree structures and progressively encoded using a thresholding mechanism that compares the magnitude of each coefficient with a predefined threshold. Coefficients below the threshold are ignored in subsequent passes, significantly reducing data volume without losing crucial information.

The statistical features—mean, variance, entropy, kurtosis, and skewness—are computed from these subband coefficients. These features are highly sensitive to the presence of epileptic seizures, which typically cause abrupt, nonlinear, and high-energy variations in the EEG signal. The EZW's capability to compress the multichannel EEG data while preserving these key attributes makes it an excellent fit for real-time, high-accuracy seizure detection systems. Additionally, EZW offers differential and cluster encoding, allowing similar segments to be grouped and encoded efficiently, further optimizing performance and bit rate. This method balances lossy compression with preservation of diagnostic quality, providing an ideal foundation for feeding machine learning classifiers such as SVM in medical diagnostic applications.

### 2.4. Cluster and Differential Encoding

The process begins by dividing each channel into *N* nonoverlapping time–domain segments, denoted as (*i*). This division is carried out in two stages for each *M* segment or *I*. In the first stage, the segments, represented as *V*(*x*), *l* = 1 ⋯ *I*. *P* are grouped based on their similarity using an association measure. The goal is to cluster segments that exhibit similar characteristics. The clustering is performed by employing a distance metric, which evaluates the proximity between segments, and then grouping these segments into clusters. The option specifies the maximum number of clusters *P*, and clusters are labeled from *p* = 1 to *P*. To refine the clustering, K-means clustering is applied. This procedure involves choosing a representative segment for each cluster based on its proximity to its center *p*. This representative segment is considered a prototype for the cluster, capturing the central tendencies of all segments in that cluster. The number of clusters *P* participates a crucial role in how closely related the segments within each cluster are. Tighter groupings within a cluster allow for more efficient encoding through differential techniques, improving data compression and accuracy. After clustering, the encoding process proceeds sequentially. Each segment *V*(*x*) is encoded progressively from *I* = 1 to *M*. Representing each segment *V*(*x*), if it belongs to a cluster *p*, then it is encoded with the cluster identifier *p* in the preamble, followed by the EZW encoded output of al_x_(*i*). In cases where *V*(*x*) does not belong to a cluster, the preamble is encoded as al_x_(*i*) and al_x_(*i*) = sp, where sp is the symbol associated with the segment, and then, the EZW output follows. The encoding process halts when the bit rate exceeds the specified threshold Nb, signaling the completion of the bitstream. This is the stop condition for the EZW encoder. Keep in mind that owing to encoding overhead, the actual bit rate can end up being higher than the planned bit rate. To ensure proper decoding and accurate reconstruction, the initial threshold values *T* and the number of segments *N* are included in an 8-bit header before each encoded segment. Finally, the bit-per-sample rate can be computed using the formula *b* + 8/*N*, where *b* represents the number of bits used for encoding and *N* is the total number of segments. The encoding process's efficiency was evaluated with respect to of information compression and bit rate with this calculation.

### 2.5. Support Vector Machine for Classification

**Figure 6:**
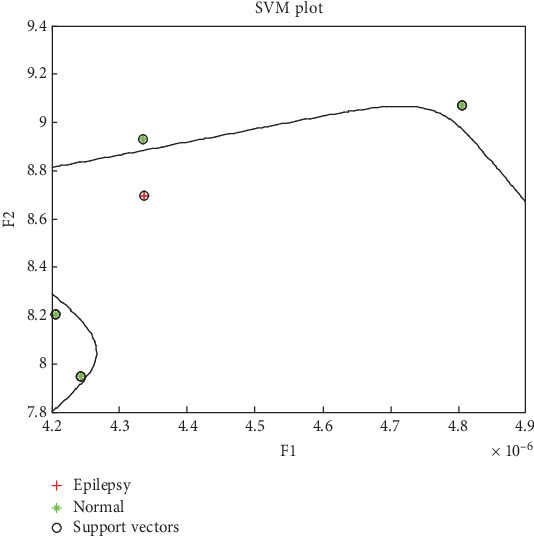
SVM-based decision boundary for signal classification.

The goal for SVM classifier's training phase is to find a decision boundary, or hyperplane, in the feature space that will divide the input data points into two independent classes [54]. Finding the optimal margin that maximizes the separation among the two groups is the major aim, as illustrated in Figure 6. Outer border of a hyperplane can be drawn by drawing two parallel hyperplanes and positioning each of them on opposite sides of the primary hyperplane. These parallel hyperplanes are equidistant from the main hyperplane and do not contain any data points. Optimal distinction among the two classes is essential, hence finding the hyperplane that maximizes margin is the goal of optimization. Optimal solutions are those hyperplanes that have the biggest training margins [56]. When deciding where to draw the line, support vectors are crucial. For the purpose of pinpointing the location of the hyperplane, these support vector data points that are geographically adjacent to the decision boundary are crucial. The key idea is that only these support vectors influence situation of the decision boundary in the feature space. In other words, feature vectors that are not support vectors do not have any impact on the placement of the hyperplane. The sample set is structured using feature vectors for each data point, denoted as *a*_1_... *a*_*x*_, associated with a class label ny. The class label indicates whether the data point belongs to one of the two classes, with *b*_*y*_ = 1 for Class 1 and *b*_*y*_ = −1 for Class 2. The decision boundary is constructed based on these class labels and feature vectors, acknowledging the SVM classifier to classify new data points into the appropriate class. The mathematical formulation of the decision boundary can be expressed as shown in Equation (4), which defines how the data points are assigned to either class based on their feature vectors:

(4) $$\begin{array}{c} D={\left\{\left(a_{x},b_{y}\right)\left|a_{x}\in R^{p},b_{y}\in \left(-1,+1\right)\right.\right\}}_{x=1}^{i}. \end{array}$$

The SVM classifier assigns each data object to one of the two categories in the feature space, based on the proximity of data point to the decision boundary. Class 1 support vectors intersect with data points that are on the same hyperplane; it is labeled with *b*_*y*_ = 1. Conversely, if the point lies on the opposite side, it is labeled with *b*_*y*_ = −1, indicating that it belongs to Class 2. The SVM classifier is taught to maximize the space between words among the two classes by determining the best decision boundary with the help of support vectors. This process ensures the classifier's ability to separate the data into two categories with maximum confidence, improving its generalization capability for new data.

## 3. Results and Discussion

**Figure 7:**
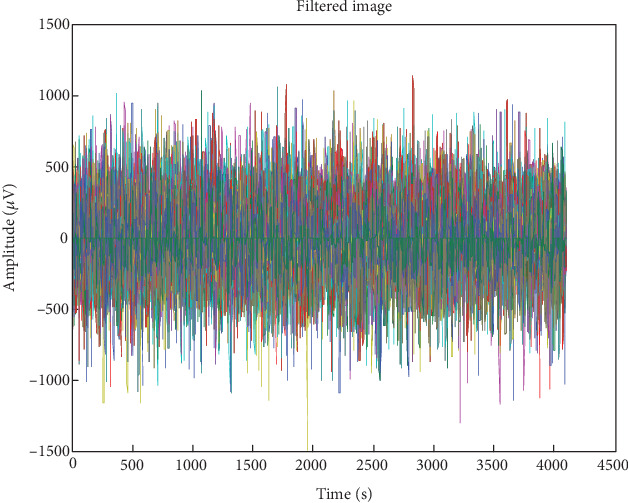
EEG signal processed with an adaptive median filter.

**Figure 8:**
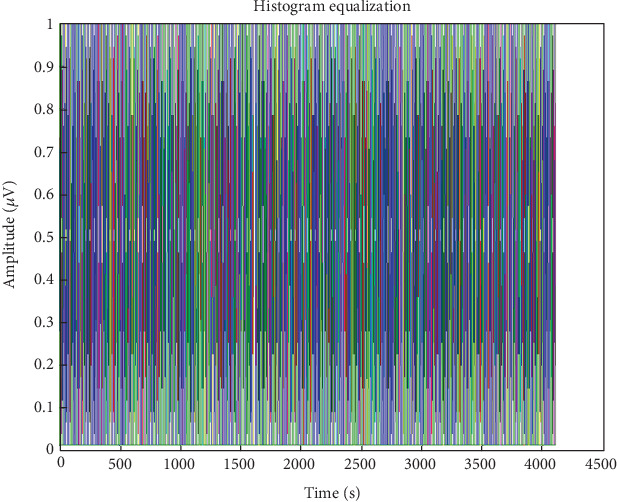
Filtered EEG signal enhanced using histogram equalization.

**Figure 9:**
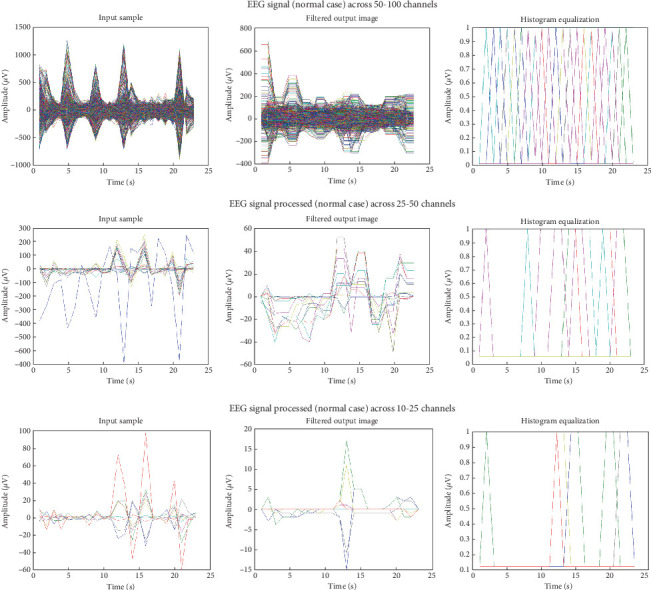
EEG signal preprocessing (normal case) across 10–25 channels, 25–50 channels, and 50–100 channels.

**Figure 10:**
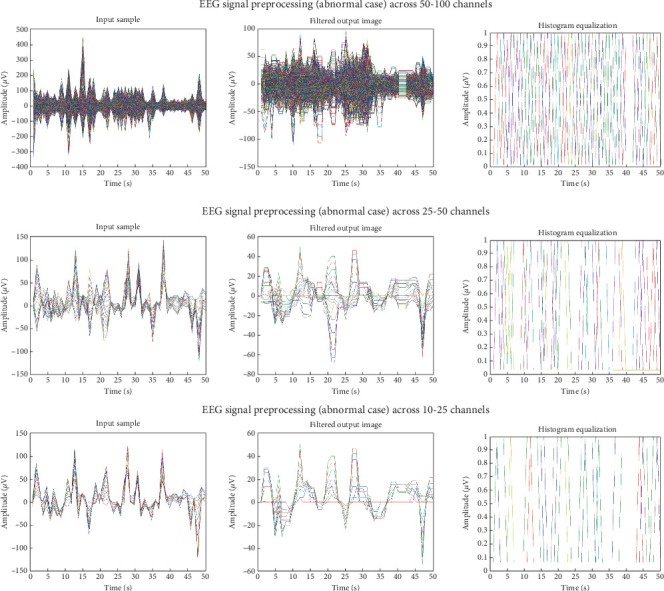
EEG signal preprocessing (abnormal case) across 10–25 channels, 25–50 channels, and 50–100 channels.

Adaptive mean filtering was used in the first preprocessing step to eliminate ocular artefacts, as shown in Figure 7. As seen in Figure 8, this procedure raises contrast in high histogram regions while lowering it in low histogram areas. Therefore, preprocessing was input data from various channels in pathological and normal participants; Figures 9 and 10 show the outcomes. These numbers show that when it comes to results, single-channel data performs better than multichannel data.

### 3.1. Metrics Analysis

**Figure 11:**
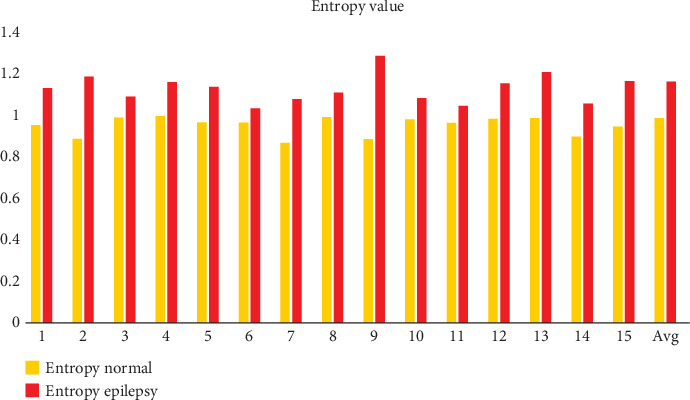
Entropy analysis for normal and epilepsy subjects.

Five statistical moments in the temporal domain were calculated for every subband to analyze and pinpoint changes across various seizure states. EEG signals from 15 different datasets were analyzed and summarized in Table 1 in the temporal domain. The five distinct qualities for which the system's overall performance measurements were calculated across various datasets were mean, kurtosis, skewness, variance, and entropy. Higher-order moments in the EEG are statistical indicators of the component dispersion and signal complexity, including kurtosis and skewness. The order of a signal is an indicator of its unpredictability. Energy entropy, a statistical measure of how the energy is distributed in an EEG signal, is given by Equations (5) and (6).  Figure 11 shows that the energy distribution of the signal's entropy is noticeably more significant than the matching control participants' entropy values:

(5) $$\begin{array}{c} \text{entropy}=\text{sum}\left(z\log_{2}z\right), \end{array}$$

(6) $$\begin{array}{c} z=\text{count of histogram}. \end{array}$$

For statistical purposes, kurtosis is essential for describing the form of a dataset's probability distribution; this is especially true when analyzing EEG signals. It provides insights into the signal's propensity to have extreme values, such as large spikes or outliers, comparative to a normal distribution. Specifically, kurtosis quantifies the “tailedness” of the distribution, which refers to the degree of concentration of data points in the tails (extreme values) compared to a normal distribution. In the case of EEG signals, kurtosis is used to evaluate the complexity and variability of the recorded data, offering valuable information about the signal's dynamic properties. In EEG analysis, a higher kurtosis value signifies that the signal has frequent and sharp peaks or outliers, meaning there are notable deviations from the signal's mean. These peaks represent significant events, such as abrupt changes in the brain's electrical activity, which are often observed in abnormal states like epilepsy. These abrupt deviations from the mean indicate a higher frequency of extreme changes in the signal, potentially corresponding to intense brain activity or seizures. A flatter and more constant signal surrounding its mean, with fewer extreme values or outliers, is indicated by a lower kurtosis value. This typically corresponds to more stable and less volatile brain activity, such as when the brain is at rest or engaged in low-frequency processes. A flatter EEG signal would have less pronounced peaks, indicating minimal variation in the brain's electrical activity. Whenever normalized by the square root of the variance, kurtosis is the fourth and central moments of a dataset, according to mathematical definition. The formula for calculating kurtosis is given by

(7) $$\begin{array}{c} \gamma =\frac{\text{Exp}\left[{\left(a\left(i\right)-\vartheta \right)}^{4}\right]}{{\left[\text{Exp}\left[{\left(a\left(i\right)-\vartheta \right)}^{2}\right]\right]}^{2}} \end{array}$$

where *ϑ* is the SD and Exp is the expected value estimator of the signal (*a*(*i*.))

**Figure 12:**
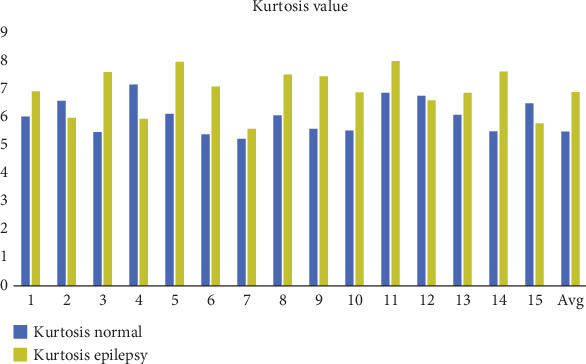
Kurtosis analysis for subjects with and without epilepsy.

The result of this calculation yields the kurtosis value. Intended for a normal distribution, the kurtosis value is typically 3, and any deviation from this value indicates a departure from normality in the data's distribution. Leptokurtic distributions (those with heavy tails and more severe peaks) are indicated by kurtosis values larger than 3, whereas platykurtic distributions (those containing lighter tails and fewer extreme peaks) are indicated by values less than 3. In the context of EEG signal processing, monitoring kurtosis values can help in identifying abnormal patterns in brain activity, such as seizures or other neurological events, which are often characterized by sharp and sudden peaks. Leptokurtic distributions (those with heavy tails and more severe peaks) are indicated by kurtosis values larger than 3, whereas platykurtic distributions (those containing lighter tails and fewer extreme peaks) are indicated by values less than 3. There was a substantial difference in the kurtosis values compared to the control participants, as shown in Figure 12.

One way to quantify the imbalance or lack thereof in a dataset's distribution is by using skewness, a statistical term. The skewness of EEG signal tells us a lot about how the signal is distributed in relation to its mean. This asymmetry can reveal important characteristics about the brain's electrical activity, which remains essential for understanding patterns in normal versus abnormal EEG signals. Mathematically, skewness is calculated as the third standardized moment of the dataset, which measures the degree to which the distribution deviates from symmetry. The formula for skewness is given by

(8) $$\begin{array}{c} S=\frac{\text{Exp}\left[{\left(a\left(i\right)-\vartheta \right)}^{3}\right]}{\rho }. \end{array}$$

*ϑ* is the mean of the dataset, *ρ* is the SD of the dataset, and E is the expected value estimator of the signal *a*(*i*).

In EEG signals, positive skewness often indicates that the signal has rare but extreme values that are higher than the typical values. These outliers may be indicative of abnormal brain activity such as spikes or seizures, which can manifest as sudden, high-amplitude signals in the EEG. In EEG signals, negative skewness suggests that the signal is dominated by smaller values, with occasional extreme low-amplitude spikes or drops that might occur in certain conditions like deep sleep or certain brain disorders. These events may be significant in detecting anomalies in brain activity, especially in diagnosing conditions like epilepsy or sleep disorders. For EEG signals, zero skewness typically indicates a balanced or normal distribution of the signal, such as during periods of calm, steady brain activity or a resting state without any significant abnormalities.

**Figure 13:**
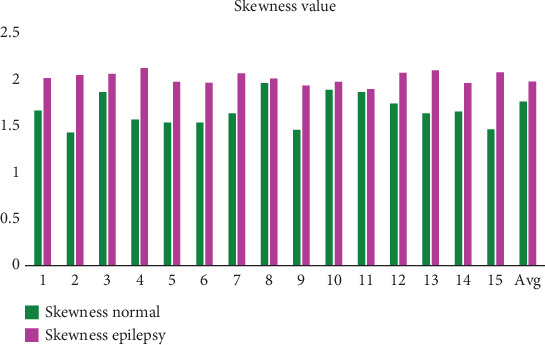
Skewness analysis for normal and epilepsy subjects.

**Figure 14:**
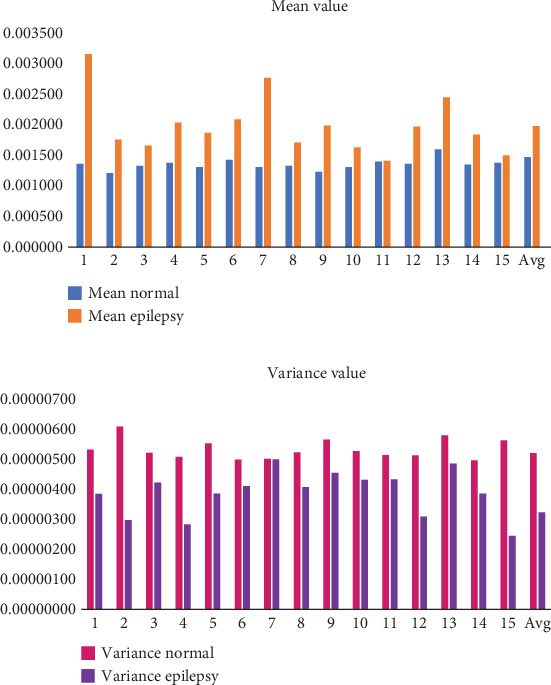
Mean and variance analysis for normal and epilepsy subjects.

The variance of a signal is a fundamental statistical measure that quantifies the degree of variation or dispersion within the signal. It provides a numerical value that reflects how much the instantaneous values of the signal deviate from the mean (average) of the signal. The variance is a key metric in signal processing and is commonly used to characterize the stability or fluctuations of a signal over time. The variance of a signal is mathematically defined as the average of the squared differences between each instantaneous value of the signal and the mean value of the signal. A signal with low variance is often considered stable and consistent. In EEG signals, this could correspond to a period of calm brain activity or a steady state, such as during deep sleep or at rest. Signals with high variance are more erratic or volatile. In EEG signals, this might represent periods of high brain activity, such as during cognitive tasks, stress, or seizures. In these cases, large deviations from the mean are indicative of sudden changes or irregularities in the signal. In the context of EEG signals, a low variance could indicate a stable baseline or background activity, whereas a high variance could signal abnormal or intense brain activity that requires further analysis. The normalization of the signal results in a lower mean for normal participants than for epileptics. Nonetheless, compared to normal participants, the variance is larger in epileptic subjects. The variance of the epilepsy data is less than predicted. In contrast, the data's mean, kurtosis, and entropy are higher than expected, as Table 2 and Figures 11, 12, 13, and 14 clearly show. On the other hand, the skewness suggests a different performance.

### 3.2. The Confusion Matrix of Seizure Detection

When human classification of data is impractical or inaccurate, machine learning steps in to fill the void. In such cases, algorithms can be trained to automatically classify instances based on input features. In datasets that are unbalanced (where one class is much more common than the other), recall and accuracy are two important measures used to evaluate the effectiveness of classification algorithms. Accuracy is the measure of how well the model predicts the future. As a whole, it is the sum of all positive predictions (both actual and hypothetical) divided by the number of actual positive results (TP). It may be stated mathematically as

(9) $$\begin{array}{c} \text{Precision}=\frac{\text{TP}}{\text{TP}+\text{FP}}. \end{array}$$

There will be fewer FPs if the model has a high degree of accuracy when predicting positive classes. The sensitivity, true positive (TP) rate, or recall of a model is a measure of its capacity to detect all significant instances in the dataset. It is the ratio of the number of actual positive outcomes (the sum of TPs and false negatives [FNs]) to the number of TP findings. In the absence of FNs, when the model correctly detects each and every occurrence of the positive class, recall can reach its maximum value of 1. Return, in mathematical terms, is defined as

(10) $$\begin{array}{c} \text{Recall}=\frac{\text{TP}}{\text{TP}+\text{FN}}. \end{array}$$

As a hybrid metric that takes accuracy and recall into account, *F*1 score provides a happy medium. The *F*1 score is maximized when both recall and accuracy are high; it is the harmonic mean of the two. In situations when the distribution of classes is not uniform, the *F*1 score can be more helpful than accuracy alone in evaluating performance, especially when the relative value of FPs and FNs is varied. The *F*1 score can be calculated using the following formula:

(11) $$\begin{array}{c} F_{\text{score}}=2\times \frac{\text{precision}\times \text{recall}}{\text{precision}+\text{recall}}. \end{array}$$

Precision and recall are essential for evaluating classification models in situations where class imbalance exists or the consequences of misclassification vary. A more comprehensive measure of a model's performance, the *F*1 score is favored for many machine learning applications, especially when it comes to medical diagnostics and real-time seizure detection. TP favorable class epochs are accurately classified. Periods that belong to the negative class are called true negatives (TNs). Epochs that pertain to the harmful category but are wrongly categorized as falling into the positive class are known as FPs and vice versa for FNs and FP. To assess how well a classification model works, the confusion matrix is essential; this is especially true when trying to identify epileptic episodes in EEG data. As a whole, it shows how well a model can tell the difference between positive and negative examples. Several important metrics are included in the matrix:

• TP: The amount of positive epileptic seizures that the model was able to identify.
• TN: The number of normal EEG patterns that were correctly identified as negative by the model.
• FP: The number of normal EEG patterns that were incorrectly classified as epileptic seizures.
• FN: The amount of FPs for normal EEG patterns caused by epileptic episodes.

In this specific study, the model assigns a value of +1 to represent epileptic seizures and −1 to represent normal EEG patterns. This binary classification helps the system differentiate between seizure and nonseizure events. The precision and accuracy of a model are two key metrics derived from the confusion matrix. Accuracy evaluates how well the model predicted overall, while precision reflects the number of positive cases (seizures) that were predicted to be really right. While precision is important, accuracy is equally critical. A model can have high precision but still be unsuitable if it lacks accuracy, meaning it could be making a large number of correct predictions in one category while failing miserably in another. In some cases, a model might achieve high precision (e.g., correctly identifying many seizures) but suffer from poor accuracy if it fails to generalize well to new data or classifies a large number of normal EEG patterns incorrectly. Conversely, accuracy alone is not sufficient to evaluate the model's ability to correctly identify seizures unless both high precision and recall (or sensitivity) are achieved. These measures, when analyzed together, give a complete picture of the model's effectiveness. The evaluation was conducted employing a leave-one-out training approach, a type of cross-validation that involves utilizing every instance in the dataset once as a test case and the rest for training. This method offers a more accurate assessment of the model's performance as it uses each of the information points to evaluate it. The proposed method outperformed previous research, as indicated by the 99 accurate predictions it made. This underscores the robustness and high performance of the SVM model for epileptic seizure detection. The detailed analysis of these results is provided in Table 3, which likely includes a more in-depth comparison between the new approach and previous techniques, showing how the proposed method improves seizure detection accuracy. By applying the formula, a considerable actual positive value indicates excellent accuracy or precision. Table 3 shows that data samples with the bulk of pertinent information are correlated with high accuracy. The recall column shows the relevant events that were correctly detected. It also shows that the recall value goes down with FP and up with FN. In a similar vein, accuracy rises as recall falls. Also, it illustrates how accuracy and precision are interdependent. The percentage of accurate predictions the model makes is known as accuracy. High precision usually translates into favorable accuracy. Stated differently, accuracy increases as the model closely approaches the maximum score. On the other hand, the model could not be considered appropriate if it lacks both precision and accuracy. Under some conditions, samples with high precision and poor accuracy may be close to one another yet far from the maximum score. Additional studies are included in Table 3 for a more thorough analysis of the research's conclusions. With 99 accurate predictions, the new study's results outperformed those of earlier investigations. The proposed method used a machine learning model termed the SVM with a 99.02% accuracy rate. It was evaluated utilizing a leave-one-out training strategy.

### 3.3. Comparison With Other Feature Extraction and Classifiers

In this study, we examined the results of EZW and SVM by examining the statistical variations between training data that were seizure-free and that were seizure-active. The detailed eigenvalue that EZW computed are shown in Table 2. The statistical study indicates that the most significant differences are in the SD and variance among seizure-free and seizure-active conditions. Even if the eigenvalue SD and variance are significantly greater, their eigenvalue mean, skewness, and kurtosis are not that different from each other. Numerous feature extraction and classification technique combinations have been investigated for outstanding accuracy in the current system. Distinct classifier types and feature extraction strategies were utilized to compute the classification accuracies for individuals with epilepsy and neurotypical people. The SVM model evaluates the effectiveness of the EZW approach, with the results presented in Table 3. As shown in Table 4, the EZW method demonstrates superior performance in real-time epileptic seizure detection, achieving higher accuracy and fewer FPs. Various feature extraction and machine learning techniques were tested and compared using evaluation data. The combination of EZW and SVM achieved an impressive accuracy of 99.02% in detecting epileptic seizures. Additionally, alternative methods for identifying EEG epilepsy signals were explored, and their results were benchmarked against the EZW-SVM model. Table 4 summarizes these findings, highlighting that the integration of EZW feature extraction with the SVM classifier achieves state-of-the-art accuracy for early epilepsy diagnosis.

The diagnosis of most brain disorders primarily relies on the manual evaluation of EEG data by neurologists or trained medical professionals. The proposed approach is aimed at assisting in the automatic identification of neurological conditions, potentially reducing diagnostic time and addressing the limited availability of neurologists. As the most intricate organ in the body, the human brain holds valuable insights into neurological disorders. Our research findings suggest that the proposed method can diagnose neurological conditions with accuracy and consistency. Integrating automated seizure detection could serve as an effective and practical solution.

## 4. Conclusion

Epilepsy is a prevalent yet poorly understood neurological disorder that severely impacts the quality of life of affected individuals. People who deal with epilepsy are always on the brink of a devastating seizure, which can cause serious harm or perhaps death. The unpredictability of these seizures makes it crucial to develop effective prediction systems. Analysis of EEG recordings may allow for early diagnosis and prediction of epileptic episodes, according to recent studies. These recordings reveal important information about the brain's electrical activity. One way to improve seizure prediction and management is to use state-of-the-art signal processing and machine learning algorithms to differentiate between seizure and nonseizure states. In order to handle and analyze EEG data, this study used supervised learning techniques. Each EEG recording is classified as a seizure or nonseizure event in supervised learning, which allows the model to be trained using labeled data. The goal is to teach the system to recognize patterns in the EEG signals that distinguish between these two states, thereby enabling real-time prediction of epileptic seizures. EEG signals represent the brain's electrical activity and are crucial in diagnosing various neurological disorders, including epilepsy, Alzheimer's disease, and autism. Recent advancements in EEG signal processing techniques have significantly improved the ability to interpret these complex signals. This is critical, as EEG recordings are a fundamental diagnostic tool for monitoring and diagnosing brain conditions. However, EEG data is often noisy and prone to various artifacts (e.g., eye blinks and muscle movements), which can obscure meaningful patterns in the signals. To ensure the quality and reliability of the EEG data, this study applies several preprocessing techniques. Real-time EEG data was recorded using the Allengers VIRGO EEG machine with a 24-channel electrode set. This high-density electrode setup allows for more comprehensive data capture from different regions of the brain, enhancing the sensitivity of the system in detecting seizures. An adaptive median filter is used to minimize noise and artifacts in the recorded EEG signals. This filter effectively reduces high-frequency noise while preserving the integrity of the signal, especially when accounting for both current and previous artifacts. This is crucial because EEG signals are often contaminated with interference from eye movements, muscle activity, or electrical noise, which can distort the true brain activity patterns. Additionally, histogram equalization is applied to enhance the contrast and dimensionality of the EEG signals. This technique adjusts the brightness and contrast levels of the signal, ensuring that the relevant features within the EEG data are more distinguishable. By enhancing the contrast of the signal, histogram equalization helps to reveal subtle patterns that might be critical for seizure detection. Once the data is preprocessed, feature extraction plays a key role in identifying the unique characteristics of the EEG signal that distinguish between seizure and nonseizure states. This study utilizes matrix decomposition through embedded EZW to extract these features. The EZW algorithm is particularly suited for compression and feature extraction because it reduces the data into a bitstream with minimal loss of information while preserving the essential features required for classification. Feature extraction is performed on key statistical metrics derived from the EEG signal, including the following:

• Mean is the average signal value over time, which provides insight into the overall activity level.
• Variance measures the signal's variability around the mean, indicating the extent of fluctuation in brain activity.
• Skewness quantifies the asymmetry of the signal distribution, with positive skew indicating that values are more spread out to the left and negative skew suggesting a right-side spread.
• Kurtosis reflects the “peakedness” or sharpness of the signal distribution. Higher kurtosis values are indicative of more pronounced signal peaks, which are often associated with seizure events.
• Entropy is a measure of the randomness or unpredictability of the signal. Higher entropy values are typically seen in seizure states due to the erratic and unpredictable nature of brain activity during a seizure.

Metric analysis of these extracted features reveals that epileptic patients exhibit greater variation, skewness, and entropy compared to healthy individuals, highlighting distinct patterns in the EEG that can be leveraged for classification. To classify the EEG signals into seizure and nonseizure categories, several machine learning classifiers are assessed, with support SVM classifiers emerging as the most effective. SVM is a supervised learning algorithm that constructs a decision boundary (hyperplane) to separate data points from different classes. The strength of SVM lies in its ability to create a hyperplane that maximizes the margin between the two classes, leading to improved generalization and robustness in classification tasks. The SVM classifier used in this study achieves a high level of accuracy in distinguishing between seizure and nonseizure states. The recovered features from the EEG data (mean, variance, skewness, kurtosis, and entropy) are fed into the SVM for training. The model is then able to accurately classify new EEG data as either a seizure or nonseizure event. The leave-one-out cross-validation method is employed to evaluate the performance of the model, ensuring that each instance of data is used for both training and testing, providing a robust measure of the model's effectiveness. The automated system developed in this study offers a high accuracy rate of 99.02% in detecting epileptic seizures, surpassing the performance of previous models. This level of accuracy is critical, as it significantly reduces the chances of misclassification, which can lead to incorrect diagnoses. Furthermore, the system operates efficiently using data from a single EEG channel, making it a viable solution for real-time epilepsy monitoring with minimal equipment. By automating the classification process, the proposed approach enables neurologists to diagnose epilepsy more rapidly and accurately. This has the potential to greatly improve the management and treatment of epilepsy, allowing for better prediction and prevention of seizures and ultimately improving the quality of life for patients living with epilepsy.

## Figures and Tables

**Table 1:** Comparison of earlier research using various datasets, feature extraction strategies, and classification approaches.

**References**	**Feature extraction**	**Classifier**	**Dataset**	**Accuracy**	**Delay**
Bhattacharyya et al. [31]	Tunable-Q wavelet transform, singular value decomposition	SVM	Z, F, N, O, S	98.6%	Not reported
Donos et al. [35]	Time domain and power bands features	Random forest classifier	European Epilepsy Database	93.84%	3.03S mean
Ahammad et al. [11]	Decomposed with Daubechies wavelet	Linear classifier	CHB-MIT database	84.2%	1.76
Shen et al. [37]	DB4-DWT	SVM	Z, F, N, O, S	96.38%	10.42
Shen et al. [37]	DB16-DWT	Rusboosted tree ensemble method	CHB-MIT	96.38%	10.42
Zabihi et al. [39]	Seven features derived from phase space and Poincare section intersection points	LDA classifiers	CHB-MIT	93.11%	Not reported
Wang et al. [40]	DTF	SVM	Ten patients with refractory epilepsy Xi'an Jiaotong University	Average detection rate of 95.89%	Not reported
Zarei et al. [42]	Orthogonal matching pursuit (OMP) techniques	Support vector machine classifier	Three widely used EEG datasets	97%	Not reported

**Table 2:** Statistical analysis in EZW method.

**Datasets**	**Entropy**		**Skewness**		**Kurtosis**		**Variance**		**Mean**	
	**Normal**	**Epilepsy**	**Normal**	**Epilepsy**	**Normal**	**Epilepsy**	**Normal**	**Epilepsy**	**Normal**	**Epilepsy**
1	0.95355	1.13147	1.66593	2.01531	6.02484	6.92364	0.00000534	0.00000386	0.00136	0.00316
2	0.88765	1.18764	1.42784	2.04524	6.58972	5.98762	0.00000611	0.00000298	0.00121	0.00176
3	0.98952	1.09162	1.86419	2.06103	5.47036	7.60605	0.00000523	0.00000423	0.00133	0.00166
4	0.99782	1.16136	1.56671	2.12408	7.16266	5.94341	0.00000509	0.00000284	0.00138	0.00204
5	0.96734	1.13875	1.53678	1.97377	6.12323	7.97487	0.00000554	0.00000387	0.00131	0.00187
6	0.96552	1.03472	1.53498	1.96254	5.39875	7.09274	0.000005	0.00000412	0.00143	0.00209
7	0.86765	1.07864	1.63553	2.06437	5.23452	5.58738	0.00000503	0.00000501	0.00131	0.00277
8	0.9923	1.1104	1.96046	2.0093	6.07054	7.52427	0.00000524	0.00000408	0.00133	0.00171
9	0.88653	1.28754	1.45624	1.93427	5.58763	7.45636	0.00000567	0.00000456	0.00123	0.00199
10	0.98147	1.08415	1.88705	1.97289	5.53008	6.88771	0.00000529	0.00000432	0.00131	0.00163
11	0.96454	1.04632	1.86536	1.89636	6.86734	8.00024	0.00000515	0.00000434	0.0014	0.00141
12	0.98337	1.15452	1.73883	2.07108	6.76489	6.60345	0.00000514	0.0000031	0.00136	0.00197
13	0.98763	1.20985	1.63554	2.09872	6.08764	6.87637	0.00000581	0.00000487	0.0016	0.00245
14	0.89765	1.05764	1.65335	1.96236	5.49874	7.62883	0.00000498	0.00000387	0.00135	0.00184
15	0.94654	1.16542	1.46378	2.07635	6.49878	5.78763	0.00000565	0.00000245	0.00138	0.0015
Average value	0.98765	1.16349	1.76235	1.97736	5.48776	6.89767	0.00000522	0.00000324	0.00147	0.00198

**Table 3:** Performance evaluation using precision, recall, and *F* score metrics for various datasets.

**Dataset**	**TP**	**FP**	**FN**	**TN**	**Precision (%)**	**Recall (%)**	**F** ** score (%)**
1	491	2	1	35	99.59	99.80	99.7
2	425	4	2	30	99.07	99.53	99.3
3	446	5	3	36	98.89	99.33	99.11
4	482	3	1	52	99.38	99.79	99.59
5	391	0	4	36	100	98.99	99.49
6	374	4	2	37	98.94	99.47	99.2
7	421	0	1	39	100	99.76	99.88
8	312	2	3	34	99.36	99.05	99.21
9	415	2	2	42	99.52	99.52	99.52
10	399	2	2	52	99.5	99.50	99.5
11	487	4	2	64	99.19	99.59	99.39
12	357	0	4	38	100	98.89	99.44
13	405	3	0	45	99.26	100	99.63
14	392	3	2	50	99.24	99.49	99.37
15	465	5	0	46	98.94	100	99.47

**Table 4:** Comparison with existing state of the art.

**References**	**Feature extraction techniques**	**Classifier**	**Accuracy**
Escobar-Ipuz et al. [7]	Extreme gradient boosting (XGB)	ML technique	98.13%
Al-Salman et al. [8]	DT-CWT and FFT based	LS-SVM	97.7%
Ahammad et al. [11]	DWT	Linear classifier	84.2%
Fergus et al. [12]	Feature-ranking algorithms and PCA	KNNC	93%
Sharanreddy and Kulkarni [15]	Multiwavelet transform	Feedforward neural network	93%
Ibrahim et al. [17]	DWT	KNN	94.6%
Lasitha [20]	Harmonic wavelet packet transform	Relevance vector machine (RVM)	99.8%
Omidvar et al. [27]	DWT	2 class SVM	98.7%
Oweis and Abdulhay [29]	Hilbert–Huang transform	Supervised Euclidean clustering	94%
Hu et al. [25]	CNN	SVM	86.25%
Bhattacharyya et al. [31]	Tunable-Q WT, SVD	SVM	98.6%
Gao et al. [33]	DWT	LDA	96.88%
Donos et al. [35]	Time domain and power bands features	RFC	93.84%
Ahammad et al. [11]	Decomposed with Daubechies wavelet	Linear classifier	84.2%
Shen et al. [37]	DB4-DWT	SVM	96.38%
Samiee et al. [38]	Novel multivariate textural SGD	SVM	74.2%
Zabihi et al. [39]	Seven features derived from the intersection points of the Poincaré section and phase space	LDA classifiers	96.38%
Wang et al. [40]	Directed transfer function (DTF)	SVM	95.89%
Zarei and Asl [42]	Orthogonal matching pursuit (OMP) techniques	SVM	97%
Dasha et al. [47]	The random forest (RF) and multilayer LSTM models	ML and DL	90.74% and 87.04%
Anuragi et al. [50]	FBSE-EWT	B-SVM and ET classifiers	98.3% and 97.8%
Anuragi et al. [57]	FBSE-EWT	LS-SVM, SVM, k-NN, and ensemble bagged tree classifiers	100% and 99.84%
**Proposed method**	**Embedded zero tree wavelet (EZW)**	**SVM**	**99.02%**

*Note:* Entries in bold indicate the proposed model.

## Data Availability

The datasets analyzed during the current study can be provided to third parties by contacting the corresponding author upon reasonable request.
